# Cost of high leverage in socially responsible firms in a linear dynamic panel model. Evidence from product market interactions

**DOI:** 10.1016/j.heliyon.2022.e09235

**Published:** 2022-04-13

**Authors:** Zeeshan Ahmed, Qasim Saleem, Muhammad Maroof Ajmal, Hajra Jameel

**Affiliations:** a Department of Management Sciences, University of Lahore, Gujrat Campus, Pakistan; b Gift Business School, Gift University Gujranwala, Pakistan; c University Institute of Management Sciences, PMAS - Arid Agriculture University, Rawalpindi, Pakistan

**Keywords:** Corporate social responsibility, Cost of high leverage, Product market interactions, Linear dynamic panel model

## Abstract

Corporate Social responsibility is the major challenge for senior management of firms and they devoted the significant resources for CSR initiatives. Despite of significant comprehension of CSR, an ongoing debate is still under consideration about its economic repercussions in terms of “do well by doing good”. Corporations are facing huge leverage cost with bad customer reputation in the product market. This study attempt to examine this phenomenon as the cost of high leverage in socially responsible firms and the product market interactions of those firms. The data is collected from 2009-2020 in linear dynamic setting for product market interaction, and two step system GMM estimation technique is applied for endogeneity concerns. The study identified that socially responsible firms are experiencing better growth in their sales in product market that maximize the financial benefits. However, the high leverage cost worsen their performance in product market because leverage is associated with some losses in market share due to unfavorable actions of competitors and customers. Socially responsible firms experience the low cost of high leverage which helps the firms to increase the performance in product market. Moreover, the corporate governance effectively devises the strategies to diminish the high leverage cost in the way towards better product market interactions. The results are conclusive across the financial crisis, firm’s classifications and different channels of firms. The study enables the holistic and broader understanding of CSR in the reduction of high leverage cost with an intention to increase the firm’s sustainability in product market. The study contributes with a view to ascertain the cost of high leverage in socially responsible firms for product market interactions of Pakistani firms in a linear dynamic panel.

## 1 Introduction

Corporate social responsibility (CSR) is one of the biggest challenging matters in the modern communal world as well as in the field of management in broad range (Khan et al., 2017). The conventional wisdom of modern corporate finance suggests to focus on shareholder’s interest and enhance the society’s welfare irrespective of stakeholder’s interest (Béenabou & Tirole, 2010). Firm’s CSR participation in recent years implies a long term orientation for strategic reasons (Yuan et al., 2020). It enhances the firm’s social capital and reputation; thereby they develop the investor’s trust on firms (Nguyen et al., 2021; Lins et al., 2017; Godfrey et al., 2009; Pevzner et al., 2015). Firms use the CSR activities strategically with the view to benefit economically and socially. Firms operating in competitive industries are socially responsible due to increase in firm value (Hawn and Kang, 2013; Fern´andez-Kranz and Santal, 2010). More importantly, CSR is conducive for firm’s propensity to extend the resources in social activities for competitive position in product market (Hoi et al., 2018; Boubaker et al., 2020; Leong and Yang, 2021).

With the passage of time as the market grows, the financial institutions work with policy makers and other private institutions for the growth of economy and to build public goodwill (Madugba and Okafor, 2016). Profitability should not be the only focus for firms; however, they try to create a balance between the business and social activities (Khan et al., 2017). The basic reason for the failure of business is the high operating cost as compared to its sales revenue and increase in customer loss ratio due to failure in production of social license (Kesto, 2017). Disclosure of CSR activities has a positive impact on the current year business profitability which is the determinant of CSR disclosure (Becchetti et al., 2014; Chan et al., 2014; Rajput et al., 2012). CSR activities may have no positive impact on short term financial performance of firms, but it definitely has an impact on long term fiscal advantages (Lin et al., 2009).

“The product market competition is the most powerful force towards economic efficiency” (Shleifer and Vishny, 1997). This competition in product market enormously affects the decisions to undertake the CSR activities (Flammer, 2015). The product market interactions and the capital structure are also widely discussed topics in research showing that the financial leverage has significant negative effect in product market performance (Opler and Titman, 1994; Campello, 2006). Customers, competitors and suppliers are known as important stakeholders for business. High leverage firms lead to losses in market share due to the unfavorable actions of these competitors and customers. Accordingly, they examine a negative relation between the engagement of CSR and cost of high leverage (Bae et al., 2019). Customers avoid purchasing from the firms that are highly levered because they thought that the firms may break the contracts with them by reducing the quality of products or by not continuing the product support. Moreover, they think that the financial problems can affect the firm incentives (Maksimovic and Titman, 1991; Kini et al., 2016; Matsa, 2011). In this way, there are more chances of default and bankruptcy in highly leveraged firms. It is difficult for these firms to stand alone with the attacks of creditors in the form of negative advertisement and can be forced to surrender (Bolton and Scharfstein, 1990; Chevalier, 1995; Telser, 1966). According to stakeholder theory, the external stakeholders play a pivotal role in achieving the goals of an organization in mitigating the costs of high leverage by improving the financial health of firms through sales. Roberts (1992) argued that the creditors might influence the CSR and the financial performance of organizations.

High leveraged costs and product market performance are well discussed in literature but a little attention is paid on how we can reduce or mitigate this cost. The external financing access and lower financing cost help in risk mitigation which boosts the CSR activities (Niu et al., 2022). The engagement in corporate social responsibility can mitigate the costs related to high leverage. The leverage cost is low for the firms that are high in corporate social responsibility (CSR) because it increases the trust between stakeholders and firm, and customers have better perceptions about the firms with high level of CSR (Hong and Liskovich, 2015; Servaes and Tamayo, 2013). Furthermore, there is a low risk for high CSR firms and they have wider investors (Hong and Kacperczyk, 2009; Waddock and Graves, 1997; ElGhoul et al., 2017). CSR helps the firms to make customers and competitors' action favorable to reduce the cost of high leverage (Bae et al., 2019). CSR practices can increase the shareholders’ value and can make good relations with them to get financial benefits. Research associated with this view examines that the established firms invest more in CSR (Ferell et al., 2016). Deng et al. (2013) suggested that the value enhancing mergers and acquisitions are taken by the firms that are high in CSR, and during the financial crises these firms perform better (Lins et al., 2017).

A growing concern among investors, enterprises, financial institutions and other stakeholders is to conduct the economic activities by the economic entities in a socially responsible manner. Corporate social responsibility is the major challenge faced by the senior management of firms (Albuquerque et al., 2019; Dunbar et al., 2020; Di Giuli and Kostovetsky, 2014), and they devoted the most of the significant resources for CSR initiatives (Deng et al., 2013). However, despite of significant comprehension of CSR, an ongoing debate is still under consideration about its economic repercussions in terms of “do well by doing good” (Ghouma et al., 2018; Jie and Nakajima, 2014; Kim et al., 2014). It is a complex multidimensional construct where each dimension entails a different role in corporate performance from the overall effect (Galema et al., 2008).

Most of the studies focused the CSR in developed countries but the research in developing and emerging economies is scant (Lins et al., 2017; Ghouma et al., 2018). Corporate behavior in terms of CSR in emerging and developing economies is greatly different from developed economies (Kao et al., 2018; Fan et al., 2011). This study attempts to fill this gap with a view to ascertain the cost of high leverage in socially responsible firms for product market interactions in Pakistan. Corporate social responsibility practices are mostly examined in Pakistan with a view of corporate philanthropy like corporations promote the welfare through welfare and charity. There is lack of knowledge about CSR engagement in Pakistan through which they can minimize the costs and maximize the benefits. The previous studies mostly focused on the costs that are imposed on high leverage in product market performance but a less focused on the mitigation of these costs. So, it becomes pertinent to focus on how we can mitigate the cost of high leverage through CSR. Various tools of CSR are taken through content analysis of financial annual reports and they assessed their impact on the cost of high leverage and sales growth of non-financial Pakistani firms. Additionally, the linear dynamic panel model is used, and two step system GMM estimation technique was applied to mitigate the concerns of endogeneity between CSR and cost of leverage.

## 2 Literature review

Corporate social responsibility (CSR) is long and widely discussed topic in research and has become very important among academicians and business executives that can be used to create competitive advantage (Bernal-Conesa et al., 2017; Skarmeas and Leonidou, 2013; Sen et al., 2016). Companies involved in CSR should act in ethical and transparent manner to create the value for them (Carroll, 1991; McWilliams and Siegel, 2001; Banerjee, 2008). Many firms focus on CSR disclosure to attract the customers and to satisfy the shareholders and stakeholders (Selcuk and Kiymaz, 2017). They accompanied to analyze the association between financial performance and CSR disclosure (El-Halaby and Hussainey, 2015; Flammer, 2015; Husted and de Sousa, 2019; Chen et al., 2018).

Firms bearing the high leverage cost do not pay the attention towards the minimization of those costs that can be minimized through CSR. CSR reduces the cost of high leverage because the involvement of high leveraged firms in CSR activities reduces the loss in market share (Bae et al., 2019). These firms should satisfy the implicit and explicit contracts with their stakeholders (Cornell and Shapiro, 1987). Leverage itself is not a big problem that creates worry among customers, but better performing firms can sustain the cost of high leverage. However, firms that are experiencing losses face serious problems (Bae et al., 2019). Literature shows that customers, competitors, suppliers and creditors can cause the decline in performance of high leveraged firms. The high product specificity customer-driven costs are higher for those firms (Opler and Titman, 1994). The customers paid a portion of amount for implicit claims for those specialized products in terms of future services. However, firms exceeding from optimal level of leverage are most likely to break the implicit contracts that enforce the customers to avoid the purchase from highly leveraged firms.

Corporate charitable contributions can influence the sales of firms as consumers’ purchase is influenced by psychological factors and social forces. They like to purchase from the firms that are involved in CSR. The high leverage costs can be reduced through CSR activities for firms experiencing higher customer driven costs. Firms can take part in charity activities, give benefits to employees and improve its product quality to win the customers. These activities increase the financial performance of firms which helps them to regain the trust in businesses that are lost due to the financial crisis (Giannarakis and Theotokas, 2011). They involve themselves in corporate social responsibility activities to improve the goodwill and attract customers to maximize the returns. Lins et al. (2017) found that the higher corporate social responsibility (CSR) firms have the higher sales, profitability and growth than the firms that invest low in CSR. The increase in level of financial leverage in equilibrium pronounces to increase the negative product market in relation to leverage (Campello, 2006). The increase in financial debt is the reason of actions that are unfavorably taken by the competitors and customers, while the moderate indebtedness is linked with improved sales performance.

CSR investment and sales are significantly and positively correlated with each other (Paul and Devi, 2016). The CSR investment and financial performance indicators revealed a positive relationship between the growth rate of sales revenue with corporate soundness and social contribution. Both soundness and social contribution showed a positive correlation with corporate value (Cho et al., 2019). McWilliams and Siegel (2001) claimed that involvement in CSR leads to increase costs beyond the company’s original management undertakings and violation of shareholders' interests. They argue that being loyal to the original purpose of maximizing the shareholders' profits is itself the fulfillment of social responsibility. Lee and Shin (2011) examined the relationship between donation expenditure and corporate value, showing that donation spending and firm value are positively related. However, after the suitable level is reached, the correlation changes to negative which they interpret basing on the agency theory. The relationship between CSR activity and sales growth in the UK retailing sector concludes the positive correlations between donations and sales revenue of retail companies in UK. It recommends that retailers' charitable behavior can increase the sales (Nyame and Ghulam, 2019). High leverage indicates that a firm is unable to generate the revenues that are sufficient to cover its expenses. However, the high leveraged firms can increase profitability by involving CSR activities that can cause an increase in future sales growth. Firms which spend more on CSR have high value. So, CSR and performance have positive and significant relationship (Babalola, 2012).

The CSR disclosure and activities strategically increase the long term operating and financial performance of corporations (Saeidi et al., 2021; Endrikat et al., 2014; Yi et al., 2021; Horv´athov´a, 2010; Fatemi et al., 2015; Deng et al., 2013; Gao and Zhang, 2015; Becchetti et al., 2013; Huang, 2021; Margolis and Walsh, 2003; Orlitzky et al., 2003; Úbeda-García et al., 2021; Nguyen et al., 2020). CSR has the direct effect on firm performance (Margolis et al., 2009; Nguyen et al., 2021; Dixon-Fowler et al., 2013; Busch and Friede, 2018; Abu Bakar and Ameer, 2011). However, CSR investments might decline the financial performance of firms (Barnett and Salomon, 2006; Surroca et al., 2010) that happens due to inevitable costs (Chen et al., 2015). Some studies identified the neutral relationship between CSR and firm performance as cost and benefits of being socially responsible offset each other (Aupperle et al., 1985; Alexander and Buchholz, 1978; McWilliams and Siegel, 2000; Iwata and Okada, 2011; Lahouel et al., 2019; Nelling and Webb, 2009).

Firms with high leverage are not able to get the resources to satisfy all the stakeholders due to financial constraints. Financially constrained firms spend less on corporate goodness (Hong and Andersen, 2011). Managers are purposely concerned with efficient rum of operation and find it difficult to concentrate on CSR activities due to financial constraints (Jensen, 2001). Firms in the competitive industries are financially constrained and undertaking the CSR activities in such a situation is questionable (Hawn and Kang, 2013). The higher competition in product market results in reduction of profit and default probability which might increase due to such financial constraints, suggesting the resource crunch are faced by the firms (Hawn and Kang, 2013). They prefer to allocate resources in CSR activities in non-competitive industries However, the market competition acts as a disciplinary mechanism to participate in CSR activities, representing market competition as disciplinary mechanism to engage in such activities (Kitzmueller and Shimshack, 2012). Therefore, in presence of product market interactions, the effect of CSR on firm’s performance is more pronounced in non-competitive industries (Gupta and Krishnamurti, 2021).

H1 Corporate social responsibility is a significant factor of reduction in cost of high leverage that improves the performance of firms in product market.

## 3 Research methodology

The rational and logical way by which research process is planned and elements of study are analyzed for data interpretation is research methodology (Upagade and Shende, 2012). The study empirically examines the cost of high leverage in socially responsible firms in product market interactions under linear dynamic panel model. The sample selection process begins with Pakistani non-financial firms over the period from 2009 to 2020. The sample is comprised of non-financial firms that are socially responsible and they have a large impact on society and the impact is more evident (Hackston and Milne, 1996). They disclose the CSR activities in their financial statements over the sample period. For this purpose, the published annual reports are the most reliable source of information that completely documented the requisite information.

### 3.1 Main variables

#### 3.1.1 Corporate social responsibility (CSR)

CSR is a multifunction activity which is measured in various studies from different categories to view on economic and social factor. CSR score is determined by adding all categories which disclose the CSR activities (Majeed et al., 2015). Data of this variable is collected from the annual reports of firms by using content analysis (Ratanajongkol et al., 2006; Hackston and Milne, 1996; Akinpelu et al., 2013). Major categories include in this study are donation, charities, health and safety, employee benefit activities and environmental protection measures etc.

• CSR Score Index

Coding keys are used to convert the contents into number. 0 and 1 are used to code these categories. 1 shows that the firm discloses the category while the numbers 0 shows that the firms do not disclose that category. Each score is added at the end to find the final CSR score of the non-financial firms. CSR disclosure is measured after adding all the categories by following formula to get the total CSR disclosure score for a particular firm.

$$\text{CSR ​Score ​}=\text{ ​}\sum \frac{d}{n}$$

In this equation, *d* is 1 if the non-financial firm disclosed the category and 0 in the case of non-disclosure. To calculate the individual firm score, each item score is added and then divided by the total number of disclosure categories. 36 items are taken, average score is found by the total number of categories disclosed by the firms to the total number of categories.

#### 3.1.2 Cost of high leverage

High leverage impacts the product market performance of non-financial firms (Opler and Titman, 1994). In equilibrium, the increase in level of financial leverage increases the negative affect of product market related to leverage (Campello, 2006). An increase in financial debt is the reason of the actions that are unfavorable taken by the competitors and customers, while the moderate indebtedness is linked with improved sales performance. In this study, the sales growth is used as a proxy to measure cost of high leverage (Bae et al., 2019).

$$Cost\;of\;High\;Levergae\;=\frac{Sales\;growth\;}{High\;leverage}$$

$$Sales\;Growth=\frac{Sales-Sales\;in\;previous\;year\;}{Sales\;in\;previous\;year}$$

High-leverage represents the firms that take more leverage to finance their activities rather than equity financing. High leverage is computed through the long-term debt ratio (Bae et al., 2019).

$$Long\;Term\;Debt\;ratio=\frac{Long\;Term\;Debt\;}{Total\;Assets}$$

If the customers leave the firm by the hunt of competitors then firms face a decrease in the growth of sales. Sales growth is basically associated with the actions of customers and also the firm’s competitors. We also follow Campello (2006) to measure the high leverage costs by using the growth of sales to high leverage.

### 3.2 Data estimation method

CSR engagement reduces the cost of high leverage and increases the financial performance in terms of sales growth by involving different channels. However, these variables of CSR and cost of high leverage are subject to the problems of potential endogeneity. Two step system GMM panel estimator is applied to deal with endogeneity issues. System GMM gives the perfect results and also improves the efficiency of estimator with lower bias and standard errors (Antoniou et al., 2006).

### 3.3 Econometric model

This model elaborates how corporate social responsibility (CSR) is related with the cost of high leverage (HLEV) and sales growth (SG) of non-financial firms in Pakistan. This study has taken on the dynamic panel model for the empirical testing of hypothesis. The dynamic panel model is suitable to remove the endogeneity problem in the model. The main concern is endogeneity bias and GMM technique is used to remove these endogeneity problems. Keeping in view the above discussion, the following empirical model (1) is developed:

(1) $$\text{Sale}{{\text{s}}_{\text{G}}}_{\text{it}}={\text{β}}_{1}\text{Sale}{{\text{s}}_{\text{G}}}_{\text{i},\text{t}-1}+{\text{β}}_{2}{\text{CSR}}_{\text{it}}+{\text{β}}_{3}{\text{HLEV}}_{\text{it}}+{\text{β}}_{4}\text{CSR}{\text{HLEV}}_{\text{it}}+{\text{β}}_{5}{\text{SIZE}}_{\text{i},\text{t}}+{\text{β}}_{6}{\text{Profit}}_{\text{i},\text{t}}+{\text{β}}_{7}{\text{Investment}}_{\text{i},\text{t}}+{\text{β}}_{8}{\text{SELLEXP}}_{\text{i},\text{t}}+{\text{ε}}_{\text{i},\text{t}}$$

Sales growth is dependent variable which is measured as growth in the current year sales based on the last year sales (Bae et al., 2019). It is assumed as the actions of competitors and customers that can cause in increasing or decreasing the sales of a firm. The lagged dependent variable is incorporated as independent to make the model dynamic. CSR is the corporate social responsibility (CSR) which is measured through different categories with a view of economic and social factor. CSR score is determined by adding all the categories which disclose the CSR activities (Majeed et al., 2015). HLEV captures the cost which is related to high leverage and measures as sales growth to high leverage (Bae et al., 2019; Campello, 2006). High leverage and is computed through long-term debt divided by total assets (Bae et al., 2019). CSR∗HLEV is the interaction of corporate social responsibility with cost of high leverage. Firm size is measured as the natural log of total assets (Izzo and Magnanelli, 2012; Bae et al., 2019). Prof is the profitability which is scaled through operating income over total assets (Bae et al., 2019). A highly leveraged firm can increase profitability by involving in CSR activities that can cause an increase in future sales. Investment is equal to the ratio of capital expenditures to total assets (Bae et al., 2019). The capital investment of firm contributes to the future sales. Selling expenses is the ratio of all expenses related to the advertising, administrative and selling to total sales (Bae et al., 2019). ε_it_ is the error term of firm i at time t.

## 4 Results and discussion

### 4.1 Descriptive statistics

Descriptive statistics shows the average behavior of variables. The result shows that data is normally distributed and there is no identification of outliers. Table 1 presents the results related to the descriptive statistics. The average behavior of sales growth is 0.0870 which shows a positive growth in sales of firms. This indicates that involvement in CSR activities increase the sales growth of firms. Corporate social responsibility index score has an average value 0.2269. The average value of cost of high leverage is 0.2572, indicating the sales growth is 25.72% of high leverage. The interaction term of CSR with cost of high leverage is showing an average value i.e. 0.0615.

**Table 1:** Descriptive statistics.

Variables	Obs	Mean	Std. Dev	Min	Max
SG	1860	0.0870	0.4936	-1.1043	3.5793
CSR	1860	0.2269	0.1972	0	0.9979
HLEV	1860	0.2572	0.1356	0.0017	0.6603
CSR∗HLEV	1860	0.0615	0.0722	0	0.5616
Prof	1860	0.0660	0.1205	-0.4622	0.5874
Investment	1860	0.5638	0.1199	0.2513	0.7994
Size	1860	6.4201	0.6756	3.7069	8.1485
Sell Exps	1860	0.0996	0.0791	0.0000	0.4668

**Note:** The above table represents the descriptive statistics of the variables. Both tails of distribution of variables were winsorized at 1% and 99% level before the submission of descriptive statistics. The values are reported about the variables sales growth, corporate social responsibility, cost of high leverage, CSR∗HLEV, profitability, investment, size and selling expense.

### 4.2 Correlation analysis

Correlation shows the direction or strength of relationship between variables. All the independent variables should be partially correlated to avoid the multicollinearity. The correlation analysis results are presented in Table 2. All the variables in this correlation analysis are partially correlated and VIF is in lower limit, therefore no multicollinearity issue in the model. It shows the rough picture of relationship between the variables and hence it is not possible to draw the conclusion based on this correlation analysis.

**Table 2:** Correlation analysis.

	SG	CSR	CSR∗HLEV	Prof	Invest	Size	Sell Exps	HLEV	VIF
SG	1.0000								
CSR	0.0049	1.0000							2.54
CSR∗HLEV	0.0108	0.4221	1.0000						1.47
Prof	0.0030	0.0887	-0.0867	1.0000					1.02
Invest	-0.0077	-0.0311	-0.0116	0.0337	1.0000				1.01
Size	-0.1059	0.0378	0.0307	0.0047	-0.0440	1.0000			1.04
Sell Exp	0.0532	0.1253	0.1416	0.0207	0.0863	-0.1832	1.0000		1.07
HLEV	0.0298	-0.1183	0.4290	-0.0835	-0.1066	-0.0065	0.0579	1.0000	2.46

**Note:** This table represents the correlation matrix between the independent and dependent variables. The correlation is among sales growth, corporate social responsibility (CSR), cost of high leverage, CSR∗HLEV, profitability, investment, size and selling expense.

### 4.3 Cost of high leverage and product market performance in socially responsible firms

This section shows the cost of high leverage and product market performance in socially responsible firms. The results related to this particular relationship are presented in Table 3. The lagged dependent variable is a noteworthy feature of dynamic panel model and its significance confirms the dynamic panel model. The CSR activities increase the product market performance of firms in terms of sales growth. An increase in CSR leads to an increase in sales (Bae et al., 2019; Babalola, 2012). Disclosure of CSR activities would likely to increase the current business profitability (Becchetti et al., 2014; Chan et al., 2014; Rajput et al., 2012). These companies act in an ethical and transparent manner to create the value in the market (Banerjee, 2008). This prediction is in support of stakeholders’ theory which predicts that CSR activities help to improve the performance of firms. Cost of high leverage deteriorates the product market performance of firms. The increase in level of financial leverage beyond the equilibrium pronounce to increase cost of high leverage, consequently the negative product market reaction (Campello, 2006). Moreover, the customers avoid purchasing from the firms that are highly leveraged because they thought these firms can break the contracts with them by reducing the quality of their products (Kini et al., 2016; Matsa, 2011; Maksimovic and Titman, 1991). Highly leveraged firms can also face attacks by their competitors such as spreading the negative information and thus facing the high cost of leverage. So, the ability to stand in these situations is difficult for them and they can be forced to surrender (Bolton and Scharfstein, 1990; Telser, 1966; Chevalier, 1995).

**Table 3:** CSR and high leverage costs.

Sales growth is dependent variable in all the columns			
Variables	Panel A	Panel B	Panel C
SG_t-1_	0.0904∗∗ (0.0318)	0.1649∗∗∗ (0.0244)	0.1372∗∗∗ (0.0263)
CSR	0.4456∗ (0.1677)	0.2314∗∗ (0.0757)	1.6095∗∗∗ (0.2362)
HLEV	-1.5238∗∗∗ (0.2083)	-0.5492∗∗∗ (0.0991)	-1.6550∗∗∗ (0.2111)
CSR∗HLEV			4.5143∗∗∗ (0.6422)
Size		-0.2791∗∗∗ (0.0142)	-0.2931∗∗∗ (0.0254)
Prof		0.4362∗∗∗ (0.0967)	0.4213∗∗∗ (0.1147)
Invest		0.1228 (0.1311)	0.4326∗ (0.1661)
Sell Exps		0.1676∗∗ (0.1849)	0.5069∗∗ (0.2441)
Const	-0.2154∗∗∗ (0.0550)	1.6949∗∗∗ (0.0991)	1.6092∗∗∗ (0.1809)
AR(1)	0.000	0.000	0.000
AR(2)	0.410	0.832	0.702
Hensen test	0.141	0.076	0.255
No of Groups	155	155	155
No of Instruments	71	114	114

**Note:** The two step system GMM results in dynamic panel model are reported in above table. The main variables are sales growth (SG), cost of high leverage (HLEV) and corporate social responsibility (CSR). Column 2 shows the results related to corporate social responsibility (CSR), cost of high leverage (HLEV) and sales growth without control variables while column 3 represent the represent the relationship along with control variables. Column 4 shows the results related to the role of CSR between cost of high leverage and sales growth along with control variables. CSR is calculated from the content analysis of financial reports by creating dummy, Prof is EBIT to total assets, financial leverage is total debt to total assets, firm size is the log of total assets, investment is capital expenditures to total assets, and selling expense is all expenses to total sales. AR (1) significance shows the prevalence of first order serial correlation and it rejects the null hypothesis i.e rejection of no first order serial correlation among error terms. However, it accepts the null hypothesis in relation to second order serial correlation AR (2) among error terms. The insignificance of Hansen/sargan test indicates that instruments are valid and are not over identified. Overall, the findings about Hansen test, AR (1) and AR (2) represents that GMM estimator and model is correctly specified and hence no specification issues. Ramsey RESET is used to identify the model linearity (i.e. model is linear in nature or not). The insignificance of Ramsey RESET test ensures the model linearity and no omitted variable bias. Furthermore, the ignorance of cross sectional dependence might lead to severe biased estimation results and the results would be biased due to the presence of cross sectional dependence. The Pesaran CD test was used to test the existence of cross sectional dependence and it is insignificant, indicating that residuals are sectionally uncorrelated. Standard errors are shown in parentheses (); ∗∗∗, ∗∗ and ∗ show the 1%, 5%, and 10% significance levels respectively.

The CSR activities lessen the cost of high leverage that increase the sales growth in product market interactions. CSR reduces the cost of high leverage because the involvement of high leveraged firms in CSR activities reduces the loss in market share (Bae et al., 2019). Though, cost of high leverage negatively affects the sales growth but the involvement in CSR activities turns it towards the increase in sales growth of firms. So, it is necessary for firms to involve themselves in CSR in order to better withstand in difficult situations. The engagement of corporate social responsibility reduces the high leverage costs because CSR is being used as a tool to minimize the costs related to the business by increasing profits. Financial institutions give loans easily at lower cost to the firms highly involved in CSR because they thought companies with high CSR activities are high in earnings and have the capacity to repay the loans and there are less chances to default of these firms (Bae et al., 2019).

### 4.4 CSR and high leverage costs: role of corporate governance (CG)

Corporate social responsibility helps the firms towards the reduction in cost of high leverage which in turn increase the growth of sales in product market. The additional controls for corporate governance are added in order to control the governance measures and their interaction with costs of high leverage. The results related to this particular relationship are presented in Table 4. It shows the estimation results in relation to the role of corporate governance and CSR towards cost of high leverage while predicting the product market interactions of firms in Pakistan. CSR and its interaction with cost of high leverage remain unchanged, however the magnitude of relationship varies with the inclusion of corporate governance attributes. The firms efficiently move towards CSR activities that help them to minimize the cost of high leverage and maximize the benefits with an increase of sales growth in product market. The study identified that corporate governance effectively increases the sales growth in product markets. The effective governance incorporates the efficient management style. They always devise the strategies that tend to increase the sales of firms. The better corporate governance effectively increases the growth of sales and reduces the variability in performance (Cheng, 2008; Dyduch and Krasodomska, 2017; Ayuso and Argandona, 2009). An efficient governance mechanism in terms of board size, board committee and compensation benefits pays attention towards the minimization of high leverage. They focus to benefit all the stakeholders such as customers which results an increase in sales growth. In general, the presence of governance attributes and CSR disclosure mitigate the high leverage cost which strengthen the firm’s position in product market performance (Dyduch and Krasodomska, 2017; Ivanisevic and Stojanovic, 2015; Javaid Lone et al., 2016; Kesto, 2017).

**Table 4:** CSR and high leverage costs: Role of corporate governance.

Sales growth is dependent variable in all the columns			
Variables	Panel A	Panel B	Panel C
SG_t-1_	0.038∗∗∗ (0.014)	0.108∗∗∗ (0.041)	-0.170∗∗ (0.080)
CSR	0.281∗∗∗ (0.050)	0.707∗∗∗ (0.150)	1.531∗∗∗ (0.312)
HLEV	-0.085∗∗∗ (0.009)	-0.042∗∗ (0.018)	-0.271∗∗∗ (0.048)
CSR∗HLEV	0.083∗∗∗ (0.013)	0.104∗∗∗ (0.016)	0.124∗∗∗ (0.036)
BS	0.300∗∗∗ (0.070)		
BS∗HLEV	0.012∗∗∗ (0.001)		
BC		0.016∗∗ (0.008)	
BC∗HLEV		0.007∗∗ (0.003)	
COMP			0.582∗∗∗ (0.1687)
COMP∗HLEV			1.618∗∗∗ (0.286)
Size	-0.193∗∗∗ (0.010)	-0.295∗∗∗ (0.031)	-0.266∗∗∗ (0.074)
Prof	0.335∗∗∗ (0.073)	0.266 (0.184)	0.225 (0.395)
Invest	-0.264∗∗∗ (0.034)	0.006 (0.125)	-0.099 (0.288)
Sell Exps	-1.112∗∗∗ (0.122)	-0.771 ∗∗ (0.363)	-1.093∗ (0.577)
Cons	1.504∗∗∗ (0.107)	1.957∗∗∗ (0.272)	1.686∗∗∗ (0.523)
AR(1)	0.000	0.000	0.000
AR(2)	0.439	0.948	0.967
Hensen test	0.141	0.033	0.053
No of Groups	155	155	155
No of Instruments	133	93	57

**Note:** The table represent the results related to the cost of high leverage in socially responsible firms and its effect on product market performance of firm with an additional control for BS - Board Size (Column 2), BC - Board Committee (Column 3), and Compensation (Column 4). The main variables are cost of high leverage (HLEV), corporate social responsibility (CSR), interaction term of cost of high leverage with corporate social responsibility (CSR∗HLEV) and sales growth (SG) of firms. BS∗HLEV is the interaction term of board size with cost of high leverage, BC∗HELV is the interaction term of board committee with cost of high leverage, Comp∗HELV is the interaction term of compensation with cost of high leverage. The control variables are firm size, profitability, investment and selling expenses. The significance of AR (1) shows the prevalence of first order serial correlation and it rejects the null hypothesis i.e. rejection of no first order serial correlation among error terms. However, it accepts the null hypothesis in relation to second order serial correlation AR (2) among error terms. The insignificance of Hansen/Sargan test indicates that instruments are valid and are not over identified. Overall, the findings about Hansen test, AR (1) and AR (2) represents that GMM estimator and model is correctly specified and hence no specification issues. The model linearity is checked through Ramsey RESET test and its significance ensured the model linearity. The Pesaran CD test was used to test the existence of cross sectional dependence and it is insignificant, indicating that residuals are cross sectionally uncorrelated. Standard errors are shown in parentheses (); ∗∗∗, ∗∗ and ∗ show the 1%, 5%, and 10% significance levels respectively.

### 4.5 Effect of CSR and cost of high leverage: role of financial crisis

We expect that effect of CSR is more pronounced during the period (2007–2009) of financial crisis (Lins et al., 2017). Fixed effect model is applied during the crisis while two step system GMM estimation technique is applied after the financial crisis. Table 5 report the estimation results in relation to the role of CSR between cost of high leverage and product market performance of firms during and after the crisis period. The findings explore the relationship that CSR activities and cost of high leverage decrease the sales growth of firms during the financial crisis. The engagement in CSR activities negatively affects the sales and performance of firms. CSR activities creates a positive image about the firms that results a decrease in the cost of high leverage which happens to increase the sales growth of firms. With respect to after financial crisis period, CSR engagement activities improve the sales growth and financial health of firms. Most of the firms started to engage themselves in CSR activities after financial crisis. This improves the financial health and attract the customers by being socially responsible firms (Giannarakis and Theotokas, 2011). The cost of high leverage negatively affects the sales growth of firms but the involvement of CSR activities increase the sales of those firms. So, it is necessary for firms to involve themselves in CSR activities that can help them better withstand in difficult situations. The financial crises brought opportunities for the firms to engage in CSR because it has a positive effect on financial health of firms (Yelkikalan and Kose, 2012).

**Table 5:** CSR and cost of high leverage: Role of financial crisis.

Dependent variable is sales growth in all the columns		
Variables	During crisis	After crisis
SG_t-1_		0.214∗∗∗ (0.037)
CSR	-0.062∗∗ (0.030)	0.234∗∗ (0.101)
HLEV	-0.010∗∗∗ (0.004)	-0.003∗∗∗ (0.001)
CSR∗HLEV	0.105∗∗∗ (0.007)	0.126∗∗∗ (0.014)
Size	0.559∗∗∗ (0.071)	0.207∗∗∗ (0.028)
Prof	-0.218 (0.150)	0.109 (0.129)
Invest	-0.053 (0.134)	-0.785∗∗∗ (0.188)
Sell Exps	1.094∗∗∗ (0.310)	-0.807∗∗∗ (0.295)
Cons	3.576∗∗∗ (0.476)	1.832∗∗∗ (0.215)
AR(1)		0.000
AR(2)		0.481
Hensen test		0.350
No of Groups	155	155
No of Instruments		100

**Note:** The above table shows the results in the context of global financial crisis by using fixed-effect model in (Column 2) during the crisis period. Column 3 shows the results related to after crisis period (Two step system GMM). Sales growth is dependent variable while cost of high leverage, corporate social responsibility and interaction term of cost of high leverage with corporate social responsibility are independent observations. Firm size, profitability, investment and selling expenses are control variables. Lins et al. (2017) is followed in this study to define the financial crisis i.e. 2008 and 2009. Ramsey RESET is used to identify the model linearity (i.e. model is linear in nature or not). The insignificance of Ramsey RESET test ensures the model linearity and no omitted variable bias. Furthermore, the ignorance of cross sectional dependence might lead to severe biased estimation results and the results would be biased due to the presence of cross sectional dependence. The Pesaran CD test was used to test the existence of cross sectional dependence and it is insignificant, indicating that residuals are cross sectionally uncorrelated. Standard errors are shown in parentheses (); ∗∗∗, ∗∗ and ∗ show the 1%, 5%, and 10% significance levels respectively.

### 4.6 Effect of CSR and cost of high leverage: role of earnings and firm size

The sample is further classified in low earnings, high earnings, small size and large size firms. Firms having the earnings and size below the median value are categorized as low earnings and small size firms while the value above the median value are categorized as high earnings and large size firms. The results between cost of high leverage and sales growth in socially responsible firms in those classifications of firms are presented in Table 6.

**Table 6:** CSR and cost of high leverage: The role of earnings and firm size

Dependent variable is sales growth in all the columns				
Variables	Low earnings	High earnings	Small size	Large size
SG_t-1_	-0.241∗∗ (0.115)	-0.433∗∗∗ (0.138)	0.0763∗∗∗ (0.0070)	0.0714∗∗∗ (0.0106)
CSR	0.216∗∗∗ (0.076)	0.814∗∗∗ (0.160)	-0.8304∗∗∗ (0.1772)	1.1456∗∗∗ (0.1396)
HLEV	-0.232∗∗ (0.104)	-0.063∗∗∗ (0.008)	-0.6237∗∗∗ (0.1389)	1.3479∗∗∗ (0.1341)
CSR∗HLEV	0.228∗ (0.120)	0.170∗∗∗ (0.039)	2.3461∗∗∗ (0.4328)	1.9503∗∗∗ (0.3665)
Size	0.215∗∗ (0.095)	0.177∗∗∗ (0.033)	-0.1159∗∗∗ (0.0231)	-0.5120∗∗∗ (0.0329)
Prof	-0.302 (0.441)	0.109 (0.130)	-0.1067∗∗∗ (0.0430)	-0.0259 (0.0696)
Invest	-0.094 (0.176)	0.140 (0.150)	-0.7936∗∗∗ (0.1172)	0.0991 (0.1709)
Sell Exps	1.802 (1.179)	0.854∗∗∗ (0.253)	-1.1903∗∗∗ (0.1830)	1.5194∗∗∗ (0.2528)
Cons	-1.264∗ (0.692)	-1.033∗∗∗ (0.198)	0.7047∗∗∗ (0.1690)	2.9178∗∗∗ (0.2394)
AR(1)	0.025	0.000	0.000	0.001
AR(2)	0.380	0.550	0.126	0.938
Hensen test	0.553	0.584	0.338	0.119
No of Groups	75	68	106	102
No of Instruments	29	43	84	84

**Note:** The above table shows the results in relation to the cost of high leverage in socially responsible firms and product market interactions. This relationship is identified in low earnings, high earnings, small size and large size firms. Firm’s earnings and size below the median value are classified as low earnings and small size firms while the above median value are categorized as high earnings and large size firms. Two step system GMM panel estimator is applied to test the hypothesis. Column 2 shows the results related to low earning firms, column 3 results related to high earnings firm, column 4 represent the estimation results about small size firms and column 5 explore the results about large size firms. Overall, the findings about Hansen test, AR (1) and AR (2) represents that GMM estimator and model is specified correctly and hence no specification issues. Ramsey RESET is used to identify the model linearity (i.e. model is linear in nature or not). The insignificance of Ramsey RESET test ensures the model linearity and no omitted variable bias. The Pesaran CD test was used to test the existence of cross sectional dependence and it is insignificant, indicating that residuals are cross sectionally uncorrelated. Standard errors are shown in parentheses (); ∗∗∗, ∗∗ and ∗ show the 1%, 5%, and 10% significance levels respectively.

**Table 7:** Estimation results between CSR and cost of high leverage across different channels.

Variables	Supplier Channel		Competitor Channel				Customer Channel		Creditor Channel	
	Accounts Payable		HHI		LI		Advertisement Exps		Interest Coverage Ratios	
	High	Low	High	Low	High	Low	High	Low	High	Low
SG_t-1_	-0.244 ∗∗∗ (0.010)	-0.182∗∗∗ (0.010)	-0.260 ∗∗∗ (0.055)	-0.106 ∗∗∗ (0.039)	-0.081∗∗∗ (0.013)	-0.220 ∗∗∗ (0.024)	0.441 ∗∗∗ (0.047)	0.065 ∗∗∗ (0.013)	0.232 ∗∗∗ (0.017)	-0.016 (0.038)
CSR	0.767 ∗∗∗ (0.129)	0.891∗∗∗ (0.058)	2.513 ∗∗∗ (0.769)	0.511 ∗∗∗ (0.158)	2.007 ∗∗∗ (0.099)	2.843 ∗∗∗ (0.496)	1.332 ∗∗∗ (0.345)	0.584 ∗∗ (0.289)	0.434 ∗∗∗ (0.128)	0.663 ∗∗ (0.333)
HLEV	-0.193 ∗∗∗ (0.072)	-0.104 ∗∗∗ (0.041)	-1.860 ∗∗∗ (0.487)	-1.182 ∗∗∗ (0.181)	-0.761 ∗∗∗ (0.089)	-2.273 ∗∗∗ (0.397)	-0.436 ∗∗ (0.204)	-2.467 ∗∗∗ (0.288)	-1.072 ∗∗∗ (0.169)	-0.596 ∗∗∗ (0.237)
CSR∗HLEV	1.762∗∗∗ (0.362)	2.863 ∗∗∗ (0.095)	6.676∗∗∗ (2.004)	1.093∗∗∗ (0.429)	5.416∗∗∗ (0.290)	3.940 ∗∗∗ (1.017)	3.642∗∗ (1.469)	4.153∗∗∗ (0.940)	1.395∗∗ (0.566)	1.758∗ (0.961)
Size	0.168 ∗∗∗ (0.012)	0.218 ∗∗∗ (0.021)	.0072 (0.032)	0.234 ∗∗∗ (0.023)	0.208 ∗∗∗ (0.016)	0.212 ∗∗∗ (0.030)	0.197 ∗∗∗ (0.073)	-0.062 (0.038)	0.304 ∗∗∗ (0.010)	0.065 ∗ (0.035)
Profitability	-0.277∗∗∗ (0.029)	-0.520 ∗∗∗ (0.039)	-0.230 (0.224)	-1.168 ∗∗∗ (0.261)	-0.302 ∗∗∗ (0.104)	0.791 ∗∗∗ (0.154)	0.047 (0.182)	0.602 ∗∗∗ (0.239)	-0.812 ∗∗∗ (0.117)	0.099 (0.196)
Invest	-0.582 ∗∗∗ (0.050)	-0.100 (0.55)	-0.119 (0.456)	0.117 (0.206)	-1.262 ∗∗∗ (0.126)	1.165 ∗∗∗ (0.203)	-1.457 ∗∗∗ (0.223)	0.337 ∗ (0.179)	-1.639 ∗∗∗ (0.085)	-0.707 ∗∗∗ (0.143)
Sell exp	-0.388 ∗∗∗ (0.091)	-1.036 ∗∗∗ (0.126)	-1.376 ∗∗∗ (0.514)	-2.327 ∗∗∗ (0.320)	1.300 ∗∗∗ (0.123)	-0.735 ∗∗ (0.308)	0.409 (0.444)	0.257 (0.328)	-0.302 (0.182)	-0.140 (0.287)
Cons	1.052 ∗∗∗ (0.076)	1.634 ∗∗∗ (0.120)	0.282 (0.334)	1.324 ∗∗∗ (0.186)	2.440 ∗∗∗ (0.150)	0.148 (0.252)	1.746 ∗∗∗ (0.563)	-0.299 (0.265)	3.002 ∗∗∗ (0.092)	-0.052 (0.250)
AR(1)	0.010	0.001	0.001	0.000	0.004	0.001	0.001	0.000	0.000	0.001
AR(2)	0.125	0.759	0.180	0.780	0.139	0.612	0.278	0.439	0.750	0.780
Hensen test	0.997	0.498	0.496	0.165	0.425	0.497	0.378	0.064	0.420	0.527
No of Groups	125	146	107	117	135	143	45	141	121	133
No of Instruments	114	100	55	67	73	65	33	81	63	79

**Note:** This table reports the results related to different channels like suppliers, competitors, customers and creditors driven costs of high leverage. The relationship between cost of high leverage and product market interactions in socially responsible firms is tested across those channels. The Significance of AR (1) shows the prevalence of first order serial correlation and it rejects the null hypothesis i.e. rejection of no first order serial correlation among error terms. However, it accepts the null hypothesis in relation to second order serial correlation AR (2) among error terms. The insignificance of Hansen/Sargan test indicates that instruments are valid and they are not over identified. Overall, the findings about Hansen test, AR (1) and AR (2) represents that GMM estimator and model is correctly specified with no specification issues. Ramsey RESET is used to identify the model linearity (i.e. model is linear in nature or not) while building the linear dynamic panel model. Ramsey RESET test insignificance ensures the linearity of model and no omitted variable bias. The Pesaran CD test was used to test the existence of cross sectional dependence and it is insignificant, indicating that residuals are cross sectionally uncorrelated. Standard errors are shown in parentheses (); ∗∗∗, ∗∗ and ∗ show the 1%, 5%, and 10% significance levels respectively.

Firms’ engaged in CSR activities experience better growth in sales. These activities creates a positive image in product market that helps them to attract more customers. They avoid purchasing from the firms that are not socially responsible. Corporate social responsibility (CSR) effect is stronger when firms are experiencing losses in their earnings (Bae et al., 2019). Firms involved in high CSR activities can attract more customers to generate more sales. Companies that are larger in size lead to more activities of corporate social responsibility and easily gains the competitive advantage (Majeed et al., 2015). However, CSR activities do not help to increase the sales of small size firms. These firms do not have a good market reputation and also do not have enough funds to invest in CSR activities. Cost of high leverage decreases the sales growth in low earnings, high earnings and small size firms. High leveraged firms bear high leverage cost which ultimately decreases their sales growth. On the other hand, it positively contributes towards the increase in sales growth of large size firms. These firms have better credit ratings in the market, and financial institutions grant them loans on easy terms and conditions. They avail the investment opportunities at low cost of capital that helps them to increase their sales and profitability. However, the involvement in CSR activities decreases the cost of high leverage which in turn increases the performance of firms in different product market interactions.

### 4.7 Effect of CSR and cost of high leverage: customer, competitor, supplier and creditor channel

This section considers the various channels that can help the socially responsible firms to minimize the high leverage cost in order to avail the better product market performance. Supplier, competitor, customer and creditor channels are used to identify the relationship between cost of high leverage and sales growth in socially responsible firms. The results related to this phenomenon are presented in Table 7. Corporate social responsibility (CSR) significantly increases the performance of firms in product market. However, the cost of high leverage declines the firm’s performance in product market across those channels. The social responsibility attracts the customers that helps them to decline the high leverage cost which resultantly increases the performance of firms in product market. Literature shows that customers, competitors, suppliers and creditors can cause the decline in performance of high leveraged firms. The high product specificity customer-driven costs are higher for those firms (Opler and Titman, 1994). The customers paid a portion of amount for implicit claims for those specialized products in connection to future services. However, firms exceeding from optimal level of leverage are most likely to break the implicit contracts that enforce the customers to avoid the purchase from highly leveraged firms. Customers and competitors play a vital role in mitigating the costs related to high leverage by improving the financial health of firms through sales. Competitor-driven costs and customers-driven costs are faced by the actions of their customers and competitors. The higher customer-driven costs have a strong effect of CSR on the growth of sales (Bae et al., 2019). Moreover, the environmental and product dimensions related to CSR have a very strong impact on the high leverage costs.

## 5 Conclusion

The study aims to find the cost of high leverage in socially responsible firms in a linear dynamic panel model in product market interactions of firms in Pakistan. The relationship is tested through the effect of CSR on cost of high leverage of non-financial firms in Pakistan in different product market interactions. The time frame is consisted upon 2009–2020. The linear dynamic panel model is developed, and two step system GMM estimation technique is applied to remove the endogeneity problems. The CSR is measured through CSR score index, and cost of high leverage is measured through the sales growth divided by high leverage. Cost of high leverage deteriorates the product market performance of firms. The increase in leverage level beyond the equilibrium pronounce to increase the cost of high leverage, consequently the negative product market reaction. The relationship of CSR with sales growth is significant and positive but the impact of high leverage cost is adverse on sales growth and it has a negative relation with sales growth. The corporate social responsibility (CSR) engagement is very important factor for firms to maximize the financial benefits, sales growth and minimizing the costs of firms. The effective corporate governance mechanism effectively mitigates the cost of high leverage which improves the product market performance with the positive increase in sales growth. It is concluded that governance attributes positively impact the level of sales growth and strengthen the CSR disclosure activities. Moreover, CSR activities actively reduce the cost of high leverage which in turn increases the performance of firms in product market during and after financial crisis. CSR activities boost the performance of firms in product market interactions across different classification of firms and channels. But high leverage cost declines the sales growth in product market in those classifications and channels. The study concluded that socially responsible firm’s experience the low cost of leverage which help the firms to increase the performance in product market interactions. The study concluded that corporate social responsibility mitigates the costs related to high leverage by influencing the behavior of customers, competitors and suppliers favorably. We further find that corporate social responsibility activities help firms to manage its stakeholders and keep their customers by generating more sales because customers pay loyal incentives to the firms that are high in CSR.

### 5.1 Implications

The study summarized a significant implication for non-financial firms, regulatory bodies, organizations and other stakeholders. The socio economic environment helps the policy makers, managers and researcher to design the important corporate issues. It helps them to have better insights about firm’s progress and performance in such a hostile competitive environment with various CSR dimensions. Decision makers can better understand that which CSR dimensions are needed to improve the firm performance. In this way, they can evaluate their position in the market in comparison to the competitors. The study enables the holistic and broader understanding of CSR in the reduction of high leverage cost with an intention to increase the firm’s sustainability in product market. Socially responsible firms can achieve more financial benefits with the creation of better awareness among customers. Due to the growing awareness regarding CSR activities in recent time, the stakeholders expect the firm to perform in socially responsible way. Under the stakeholder theory, all stakeholders have a right to know about all the environmental implications and social activities of firm at all times (Omran and Ramdhony, 2015). Customer and competitor channels have a negative effect on the costs of high leverage but involvement in corporate social responsibility can cause the mitigation of those costs (Bae et al., 2019). CSR implementation enhances the customer satisfaction, business growth and lower the leverage cost, promoting the firm’s non-financial performance. It enlarges the firm reputation internally and externally which demonstrates the practical benefits of CSR to businesses.

### 5.2 Limitations and future research directions

Every research is coupled with number of limitation factors. The first limitation is that most of the non-financial firms in Pakistan are not rated on the basis of CSR activities that makes it difficult to find the requisite information. The financial and non-financial data is taken from published annual reports where CSR disclosure might provide the wrong information. The study relied on secondary data but did not conducted any interview or survey, it is observed that the many firms are not properly disclosing their CSR activities. The study provides a benchmark for future studies like CSR activities and firm value with an onset of pandemic. They can investigate the undiscovered aspects of CSR that are needed to reassess in a more practical way. Moreover, the social and environmental factor vary over time due to the development of economy; therefore, a longitudinal research is crucial to undertake the comprehensive information. Future research could further investigate the effect of CSR on firm’s value by extending the analysis to a wider set of stakeholders such as employees, suppliers, community, and the government.

## Declarations

### Author contribution statement

Zeeshan Ahmed: Conceived and designed the experiments; Analyzed and interpreted the data; Wrote the paper.

Dr. Qasim Saleem contributed in the following ways like Theoretical alignment of subject, data analysis, final writing of article and proof reading.

Hajra Jameel: Performed the experiments.

Muhammad Maroof Ajmal: Contributed reagents, materials, analysis tools or data.

### Funding statement

This research did not receive any specific grant from funding agencies in the public, commercial, or not-for-profit sectors.

### Data availability statement

Data will be made available on request.

### Declaration of interests statement

The authors declare no conflict of interest.

### Additional information

No additional information is available for this paper.
